# An integrated meta-analysis approach to identifying medications with potential to alter breast cancer risk through connectivity mapping

**DOI:** 10.1186/s12859-017-1989-x

**Published:** 2017-12-21

**Authors:** Gayathri Thillaiyampalam, Fabio Liberante, Liam Murray, Chris Cardwell, Ken Mills, Shu-Dong Zhang

**Affiliations:** 1 Centre for Cancer Research and Cell Biology (CCRCB), Queen’s University Belfast, Belfast, UK; 2 Centre for Public Health, Queen’s University Belfast, Belfast, UK; 3 Northern Ireland Centre for Stratified Medicine, Biomedical Sciences Research Institute, University of Ulster, C-TRIC Building, Altnagelvin Area Hospital, Glenshane Road, L/Derry, Northern Ireland, BT47 6SB UK

**Keywords:** Connectivity mapping, Differentially expressed genes, Gene signature progression, Disease inhibitory compounds, Breast cancer

## Abstract

**Background:**

Gene expression connectivity mapping has gained much popularity in recent years with a number of successful applications in biomedical research testifying its utility and promise. A major application of connectivity mapping is the identification of small molecule compounds capable of inhibiting a disease state. In this study, we are additionally interested in small molecule compounds that may enhance a disease state or increase the risk of developing that disease. Using breast cancer as a case study, we aim to develop and test a methodology for identifying commonly prescribed drugs that may have a suppressing or inducing effect on the target disease (breast cancer).

**Results:**

We obtained from public data repositories a collection of breast cancer gene expression datasets with over 7000 patients. An integrated meta-analysis approach to gene expression connectivity mapping was developed, which involved unified processing and normalization of raw gene expression data, systematic removal of batch effects, and multiple runs of balanced sampling for differential expression analysis. Differentially expressed genes stringently selected were used to construct multiple non-joint gene signatures representing the same biological state. Remarkably these non-joint gene signatures retrieved from connectivity mapping separate lists of candidate drugs with significant overlaps, providing high confidence in their predicted effects on breast cancers. Of particular note, among the top 26 compounds identified as inversely connected to the breast cancer gene signatures, 14 of them are known anti-cancer drugs.

**Conclusions:**

A few candidate drugs with potential to enhance breast cancer or increase the risk of the disease were also identified; further investigation on a large population is required to firmly establish their effects on breast cancer risks. This work thus provides a novel approach and an applicable example for identifying medications with potential to alter cancer risks through gene expression connectivity mapping.

**Electronic supplementary material:**

The online version of this article (doi:10.1186/s12859-017-1989-x) contains supplementary material, which is available to authorized users.

## Background

Breast cancer is the most common cancer in England with over 46,000 women diagnosed each year [1]. It has a marked impact on mortality with relative survival rates of 80% at 5 years and 70% at 10 years [2]. These incidence and mortality rates highlight the need for additional prevention and treatment strategies for this disease.

In the UK the population is increasingly exposed to prescribed medications [3] which may have unrecognized beneficial or harmful pleiotropic effects [4]. Recently there has been much interest in exploring new therapeutic uses for existing drugs [5]. Aspirin, for example, has been shown to prevent colorectal cancer in high risk patients [6] and trials of aspirin to treat colorectal cancer are underway [7]. Similar opportunities remain to be identified for breast cancer. The potential adverse effects of common medications on breast cancer risk and progression are also worthy of investigation.

Given the health care burden/need in relation to breast cancer as described above and similarly for many other types of cancers and chronical diseases, it would be highly desirable to be able to screen systematically the commonly prescribed medications for their potential effects on altering the risk of certain disease. Furthermore, modern high throughput omics technologies and the vast volume of data generated from these technologies have provided invaluable resources for data-rich research. In this work, we aim to develop a systematic approach to utilizing the massive gene expression profiling data available for a particular disease, employing and developing gene expression connectivity mapping procedures to screen commonly prescribed medications for their potentials to alter the disease risk. By altering the disease risk, we broadly mean that the medication is able to inhibit/enhance the disease state or to decrease/increase the chance of an individual developing the disease as compared to without taking the medication. In principal, candidate medications predicted to affect disease risk could be further investigated in large population-based studies.

Connectivity mapping [8–11] is an advanced bioinformatics technique that establishes connections among different biological states via their gene expression profiles/signatures. The underlying premise of connectivity mapping is that different biological states can be adequately described or characterized using a molecular signature, such as a transcriptome, and that connections between different biological states can be established based on gene-expression similarity or dissimilarity. Connections between biological states may have different implications, for example, if a connection is seen between two states because the key set of genes are similarly up- or down-regulated, often referred to as a “positive connection”, this indicates that the two states have the same activated biological processes or pathways. On the other hand if the connection occurs because the key set of genes are oppositely regulated, referred to as a “‘reverse connection”, it may indicate that the two states negate each other. If one is an undesirable state such as disease and the other is a drug-induced state, in the former case of “positive connection” the drug might be reasonably considered to potentially induce/enhance the disease, and in the latter case of “reverse connection”, the drug may be useful to treat that particular disease.

The connectivity mapping process involves three key components: (i) A gene expression signature for a particular biological state of interest; (ii) A large reference database of differential gene-expression profiles, e.g. for a collection of small molecule compounds; (iii) A computational and statistical algorithm for matching up the gene signature and the reference profiles.

An important aim of connectivity mapping is the identification of small molecule compounds capable of inhibiting a disease state in drug discovery or repurposing research [8, 12, 13]. Connectivity mapping has been used to successfully identify medications with anti-cancer properties. For instance, cimetidine has been identified as a potential treatment for lung cancer and pre-clinically validated using mouse models [14] and rapamycin has been shown to overcome dexamethasone resistance in acute lymphoblastic leukemia (ALL) [8]. Furthermore, our research team has used the connectivity map approach to predict and subsequently validate, in a mouse model, entinostat as a potential inhibitor of acute myeloid leukaemia (AML) [15]; and recently to successfully identify and validate bromocriptine, a dopamine agonist, as a novel therapy for high-risk myelodysplastic syndromes and secondary acute myeloid leukemia [16].

In this work, we choose breast cancer as the disease of interest for our case study. This was primarily because the availability of gene expression profiling data for this disease. On the Gene Expression Omnibus (GEO) database, for example, the number of samples returned with the search term “breast cancer” far exceeds that for any other types of cancers or any other diseases. Our plan was to assemble as broad as possible many breast cancer datasets in order to derive high-quality, highly representative gene expression signatures for this disease. However, most breast cancer datasets do not contain normal controls. Therefore, the multiple dataset meta-analysis method we developed previously [17] would not be applicable, because it conducts differential expression analysis (requiring both normal and disease samples) within each dataset, and then combines lists of differentially expressed genes (DEGs) using normalized and signed ranks. Here we need to pool all the normal control samples together. Consequently comes the need to remove batch effects from the datasets and to deal with overall imbalanced sample sizes. In this work, we aim to develop a novel systematic procedure to address all these data processing and analysis challenges presented. Also we present novel connectivity mapping process using non-joint sub-gene signatures for the same disease state. This enhances the robustness of any candidate drugs returned. Such an integrated approach would also enable us to deal with similar situations arising in other studies and to facilitate the screening of medications through connectivity mapping.

It should be noted that breast cancer like many other diseases is itself a heterogeneous disease with different subtypes. In recent years there have been a lot of research efforts to classify breast cancer patients into different subtypes based on their gene expression patterns [18–20]. In this study, however, while recognizing the heterogeneity of the disease we are treating all breast cancers as a whole and focusing on the commonality rather than the finer difference between different subtypes, based on the following rationales: Firstly, there is still great value in studying the common gene expression signature of a disease, even though it consists of different subtypes. Secondly, if any of the predicted medications were to be validated, the number of patients eligible to include in future population-based studies is often a limiting factor, due to health care data availability, accessibility, and ethics etc. Focusing on individual subtypes of a disease is going to limit the sample size even further. Thirdly, even if we had focused on specific subtypes of breast cancer, and obtained candidate drugs for the subtypes. The information on the subtype of a patient’s breast cancer is often not readily available in their health care records.

## Methods

To apply gene expression connectivity mapping to breast cancer, we need gene signature(s) representing the breast cancer disease state as input. In this context, a gene signature is a selected list of genes that are differentially expressed in the breast cancer state with reference to normal condition. Breast cancer gene expression datasets were retrieved from public databases; the dataset and sample selection process is described as follows.

### Selection of datasets and samples

Gene Expression Omnibus (GEO) and ArrayExpress are public repositories of gene expression datasets that are in compliance with the Minimum Information About Microarray Experiment (MIAME) community standard [21]. GEO currently contains data on over 1 million individual samples from over 41,000 series/studies.

An explicit search through GEO and Array express using the search term ’breast cancer’ resulted in 467 data sets and the relevance of the samples were confirmed through a manual examination. The selected datasets contained samples with the following properties.

- Search Term : Breast Cancer
- Array Platform : GPL96 (Human Genome U133A Array) or GPL570 (Human Genome U133 Plus 2.0 Array)
- Population : All
- Subtypes : All
- Tissue type : Primary
- Sample size : > 20

**Fig. 1:**
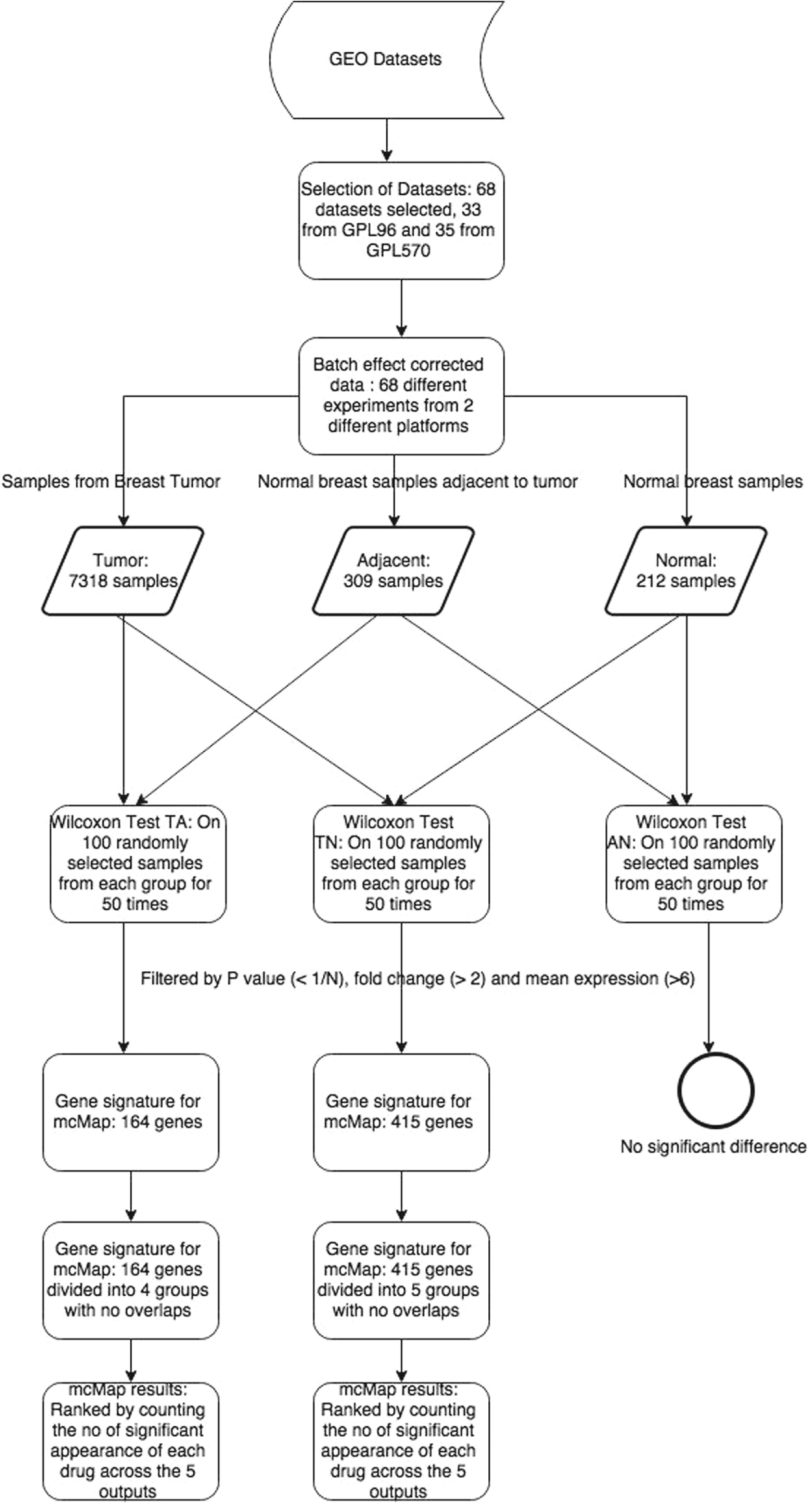
The flowchart of the process involved in this study

The GEO DataSets was searched using “Breast Cancer” as the primary search term and the results were further filtered for platforms GPL96 (Affymetrix Human Genome U133A Array) and GPL570 (Affymetrix Human Genome U133 Plus 2.0 Array), as these two platforms are compatible with the reference profile databases in connectivity mapping. The reference profiles in the CMap02 (Connectivity Map Build 02) and LINCS (Library of Network-Based Cellular Signatures) databases use the same set of gene probe identifiers as the GPL96 and GPL570 array platforms, therefore there would be no need to convert gene IDs. In total 467 datasets were retrieved, consisting of 115 individual data series from GPL96 platform and 352 from GPL570 platform. As another filtering criterion, data series with < 20 samples were excluded, which resulted in 50 datasets of GPL96 platform and 54 datasets of GPL570 platform remaining for further detailed review. For each of the 104 individual data series, their experimental design and sample description were manually examined. Finally 68 datasets in total including 33 data series from GPL96 and 35 data series from GPL570 were selected for the current study. The chosen datasets comprised gene expression data regardless of the type of breast cancer they developed and from various populations around the world. Eligible samples were categorised into three distinct groups. Tumor: Pre-treatment primary breast tumor samples. Normal: Breast tissue samples from healthy individuals with no history of breast cancer. Adjacent: Healthy breast tissue samples adjacent to tumor from breast cancer patients The number of samples categorised under three distinct groups Tumor, Normal and Adjacent are 7318, 212 and 309 respectively. Figure 1 shows a flowchart of the process involved in this study and the comparisons made among the sample groups. Table 1 summarises the total numbers of samples belonging to different groups and platforms. More detailed descriptions of selected datasets are provided as supplementary data (Additional file 1).

**Table 1:** Summary of the selected samples used in this studies from two microarray platforms and three sample groups

	Tumor	Normal	Adjacent	Total
GPL96	3990	33	112	4135
GPL570	3328	179	197	3704
Total	7318	212	309	7839

### The processing of gene expression data

**Fig. 2:**
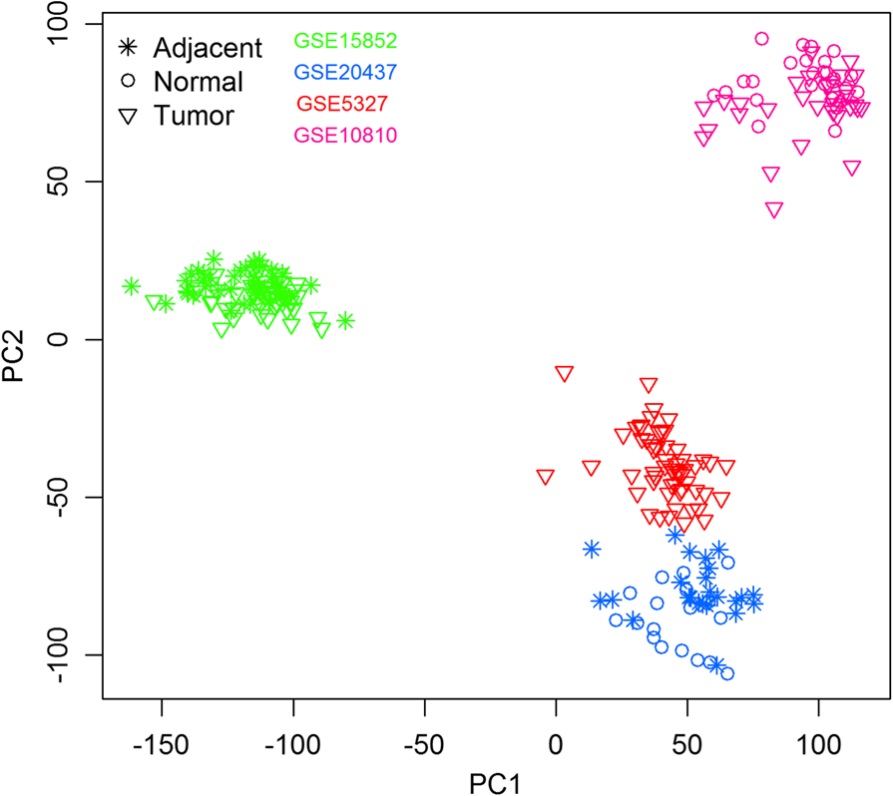
The PCA plot before batch effect removal. Three types of samples from 4 different datasets are shown on this figure; different colors indicate different datsets, while different symbols represent sample types (Normal, Tumor, or Adjacent Normal)

**Fig. 3:**
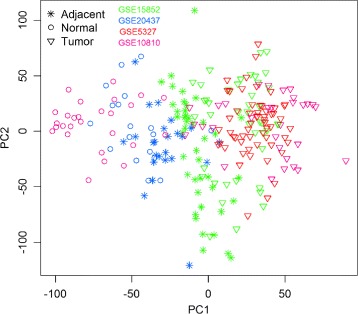
The PCA plot after ComBat batch effect removal. The same set of samples as in the previous figure, but after the ComBat batch effect removal procedure has been applied. Color and symbol schemes remain the same

The raw data CEL files of all 68 selected data sets were downloaded and a unified pre-processing and normalization method was applied. The Affymetrix MAS5 (Microarray Suite 5.0) algorithm, as implemented in the Bioconductor package affy, was applied to these microarray raw data CEL files to generate an expression data matrix for each of the 68 datasets individually. The MAS5 expression values were then transformed to a logarithmic scale of base 2, and all subsequent analyses were performed on the log2 transformed MAS5 data. The GPL96 platform contains 22283 unique Affymetrix probesIDs, while the GPL570 platform contains 54675; the number of common probeIDs between the two platforms is 22277. The 68 data matrices were finally merged into a single expression data matrix using the common probeIDs. While this increases the statistical power for subsequent differential gene expression analysis, combining datasets from different studies does present the issue of data heterogeneity and possible batch effects, which, if not properly addressed, will adversely affect all subsequent analysis and results. Figure 2 is a PCA (Principal Component Analysis) plot of the three types of samples: Normal, Tumor, Adjacent Normal, from four different datasets GSE15852, GSE20437, GSE5327, and GSE10810. As can been seen from this figure, the differences between different datasets are more pronounced than the differences between different types of samples. As we are primarily interested in the differential gene expression between sample types, this obvious “batch effect” must be removed in order to obtain meaningful results. For data integration, we employed a widely used batch effect correction method Combat [22] as implemented in the R package sva [23] to remove these batch effects. It allows user to specify for each sample its type and batch, then systematically partition the variations into two parts and remove the effects associated with batches, but retain the variation due to sample types. Figure 3 is a PCA plot of the same set of samples after the ComBat batch removal procedure has been applied. In our analysis, we applied ComBat batch removal procedure to the merged single expression data matrix described above. As a result of the data processing procedures described above, we have a MAS5 normalised, log2 transformed, and batch effect corrected gene expression matrix of 22277 genes by 7839 samples of three groups: 7318 tumor samples, 212 Normal samples, and 309 Adjacent normal samples. This gene expression matrix serves as input to our subsequent differential gene expression analysis.

### Differential expression analysis and filtering

Differential expression analysis comparing designated groups was performed to identify differentially expressed genes between these different biological states. Selecting an appropriate method to assess the extent of differential expression and the correction for multiple testing are the main issues in differential expression analysis. The differential gene expression between two given states was assessed both statistically and biologically. First, the statistical significance of any differential expression was assessed using the non-parametric two-sample Wilcoxon test. A stringent *p*-value threshold taking into account multiple testing was used to declare statistically significant findings. In this study, the *p*-value threshold is generally set as 1/*N*, where *N* is the number of genes under consideration, which is also the number of hypotheses being simultaneously tested in an analysis. This setting of threshold will control the expected number of false positive findings to be 1 in such an analysis, meaning that among the genes declared as statistically significant, on average 1 of them is expected to be a false discovery. We note here that in the classical Bonferoni method for multiple testing, the threshold *p*-value is set at *α*/*N*, to control the family-wise error rate (FWER), to be no greater than *α*, where FWER is the probability that at least one false positive error is made, and the value *α*=0.05 is often used to follow historical convention. However, the Bonferroni method is too conservative and leads to high rate of false negatives. In recent years, the FDR (false discovery rate) associated approaches have become popular in addressing the multiple testing problems encountered in the high throughput omics era. Instead of controlling FWER, the FDR approaches aim to control the rate of false discoveries, or directly the expected number of false discoveries. Our previous work carefully examined the relationships among different variants of FDRs and the advantages of eFDR (empirical FDR) over other variant FDRs were also explained [24]. From the prospective of the Bonferoni method, our *p*-value threshold of 1/*N* controls the Family-wise error rate to be no greater than 1. This simply means that among the genes that we declared as significant, it is almost certain that at least one gene will be false positive discoveries. On the other hand, the Bonferoni method with the threshold of *α*/*N* controls the expected number of false discoveries to be *α*. Therefore, one can view the same method from different angles, hence emphasizing different aspects of the same outcome.

Following statistical significance tests, genes that passed the statistical significance filter are then further examined on their magnitude of differential expression to make sure they are also biologically significant. This is achieved by calculating the gene expression fold change (log2 fold change in this study) between the two groups being compared, and with two further filters applied: 1) a gene must have a differential expression of log2 fold-change no less than 2; 2) the mean expression value of a gene must be greater than 6 (on the log2MAS5 scale) in at least one group. This means if a gene’s mean expression values are below 6 in both groups being compared, this gene will not be considered further, because of its overall low expression level. This minimum value 6 for log2MAS5, although somewhat arbitrary, was based on our extensive experience dealing with microarray gene expression data. The rationale of this filtering was that for genes with low expression levels in both conditions, we were less confident about their differential expression status, and also because of their low expression levels, their biological significance was considered less important than those with higher expression.

### Gene signature creation and connectivity mapping

All the significant genes qualified through the stringent filtering criteria described above were then sorted by combining their *p* value and fold change rankings. Briefly, the genes were initially ranked by *p*-value and by absolute log2 fold change separately, so each gene was assigned two ranks, and then the average of the two is the single combined rank for that gene. After that, the genes were then ordered by this combined rank. Ordered list of genes identified as statistically and biologically significant then served as input to connectivity mapping analysis to identify drugs that can potentially alter the expressions of the signature genes and therefore increase/reduce the risk of developing breast cancer.

Gene expression connectivity mapping analyses were performed using our recently developed QUADrATiC system [13], which is a scalable gene expression connectivity mapping framework for repurposing Food and Drug Administration (FDA) approved drugs. QUADrATiC takes advantage of the multiple processor cores available in most modern desktop computers to achieve a high performance and scalable solution to computing loads in connectivity mapping. The database of reference profiles used in QUADrATiC were built from the LINCS data, with over 83,000 reference profiles for over 1300 FDA approved drugs. Each of the gene signatures compiled in the previous steps was used as an input to query QUADrATiC, which returns the connection scores and *p*-values for 1349 FDA drugs. These connection scores and *p*-values indicate how strong and significant the corresponding drugs were connected to the input gene signature. Here too, a stringent threshold *p*-value of 1/1349≈7.4×10^−4^ was used to declare significant drug-signature connection. While the *p*-value determines the statistical significance of the drug’s connection to the gene signature, the sign of the connection score informs whether the drug can potentially enhance or suppress the gene signature representing the breast cancer disease state.

## Results

Gene expression data from all 68 datasets which passed the selection criteria were used in this study. Table 1 summarises the information on datasets used and the numbers of samples belonging to three groups: Tumor, Adjacent and Normal. As a result of combining all 68 data sets, batch effect corrected log2 gene expression values were generated comprising three groups of samples: tumor (7318 samples), normal (212 samples) and adjacent (309 samples).

### Filtering and selection of significant genes

Three distinctive pair-wise comparisons were performed in differential gene expression analyses: Tumor Vs Normal, Tumor Vs Adjacent and Normal Vs Adjacent. Because of the imbalance of the numbers of samples for the three groups, a sampling procedure was adopted for the differential expression analysis. This sampling procedure results in more balanced sample sizes when comparing two groups. Based on our preliminary power calculations (see Additional files 2 and 3 for more detailed description and results of our power calculations), 100 samples per group would give sufficient power to detect differentially expressed genes. In our analyses, for each of the pair-wise comparisons, two-sample Wilcoxon test was performed on 100 randomly selected samples from each groups, and applied to each gene individually. The results of this simultaneous multiple hypothesis testing include 22277 *p*-values indicating the level of statistical significance for each gene. Any gene with a *p*-value less that the threshold 1/*N*=1/22277≈4.5×10^−5^ is declared as statistically significant. Following through the procedure, a list of significant genes can be obtained for each run of such two-group 100-vs-100 comparison.

For the Tumor vs Normal comparison, we repeated the sampling and testing procedure 50 times. Each time the samples were selected randomly from the chosen groups. As a result 50 sets of *p* values were produced and the genes that were significant across all these 50 runs were selected for further analysis because of their consistency. The numbers of statistically differentially expressed genes for the three types of comparisons are:

- Tumor Vs Normal : 3934
- Tumor Vs Adjacent: 2140
- Adjacent Vs Normal: 598

After the statistical testing, the two further filters described in the “Methods” section were applied, namely (a) the differential expression of log2 fold-change is no less than 2; and (b) the mean expression value in at least one groups is above 6. The three step filtering of significant genes resulted in the following number of genes as statistically and biologically significant.

- Tumor Vs Normal : 415
- Tumor Vs Adjacent: 164
- Adjacent Vs Normal: 4

**Fig. 4:**
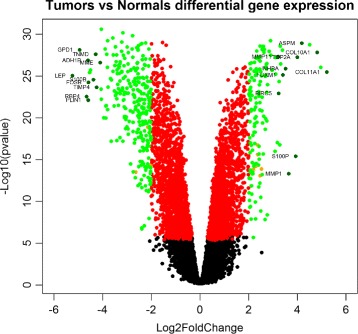
The Volcano plot of differential gene expression tumor vs normal comparison. Genes are plotted in different colors depending their passes of the following filters. Filter 1: the differential expression of gene is statistically significant, ie. *p*-valve < 1/22277, across all 50 runs; Filter 2: The absolute value of the average log2 fold change across the 50 runs is greater than 2; Filter 3: The average expression level of tumor group or normal group is greater than 6. Green spots represent genes that have passed all the 3 filters and been selected into the gene signature; Black spots represent genes that did not pass filter 1; Red: genes that passed filter 1 but not filter 2; Orange spots are genes that passed filter 1 and 2, but not filter 3. Additionally, a number of top up-regulated genes and down-regulated genes are plotted in darker green with their gene symbol as textual label. These probes are primarily selected by their magnitude of differential gene expression while avoiding label overlaps on the plot

**Fig. 5:**
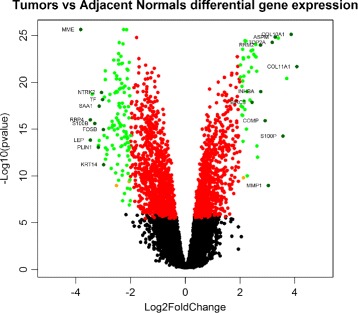
The Volcano plot of differential gene expression tumor vs adjacent normal comparison. Genes are plotted in different colors depending their passes of the following filters. Filter 1: the differential expression of gene is statistically significant, ie. *p*-valve < 1/22277, across all 50 runs; Filter 2: The absolute value of the average log2 fold change across the 50 runs is greater than 2; Filter 3: The average expression level of tumor group or normal group is greater than 6. Green spots represent genes that have passed all the 3 filters and been selected into the gene signature; Black spots represent genes that did not pass filter 1; Red: genes that passed filter 1 but not filter 2; Orange spots are genes that passed filter 1 and 2, but not filter 3

Figure 4 shows the results of differential gene expression analysis of the Tumor vs Normal comparison, with the 415 selected gene probes plotted as green dots. The full list of these 415 gene probes can be found in Additional file 4. Figure 5 shows the results of differential gene expression analysis of the Tumor vs Adjacent Normal comparison, with the 164 selected gene probes plotted as green dots. The full list of these 164 gene probes can be found in Additional file 5. Comparing the results above, there is a big overlap between the Tumor-vs-Normal 415 probes and the Tumor-vs-Adjacent 164 probes. In particular 145 out of the 164 probes (88%) are part of the 415 probes. This suggests that the adjacent normal tissue is actually very close to the normal tissue, consistent with the fact that there are only 4 probes selected in the Adjacent-vs-Normal differential expression analysis above.

In the two figures above, a number of top up-regulated and down-regulated probes are also plotted in darker green with their gene symbol shown as textual labels. These genes are highlighted (labeled) primarily based on their magnitude of differential gene expression, while avoiding label overlaps on the plots where possible. It appears that a number of the these genes are well known for their involvement in cancer. For example, BIRC5 is a member of the inhibitor of apoptosis (IAP) gene family encoding negative regulatory proteins that prevent apoptotic cell death. Its gene expression is high during fetal development and in most tumors, but low in adult tissues. This is consistent with our results here that BIRC5 as one of the most up-regulated genes in breast cancers. The top up-regulated gene with the highest magnitude of differential expression in both figures, COL11A1, has been reported to be over-expressed in recurrent non-small cell lung cancer [25] and in gastric cancer tissues [26] and to promote cell proliferation, migration, invasion and drug resistance. The over-expression of this gene has also been implicated in breast cancer progression in facilitating the transition from ductal carcinoma in situ to invasive ductal carcinoma [27]. On the other side of the volcanos, PLIN1 is one of top down-regulated genes in both our Tumor-vs-Normal and Tumor-vs-Adjacent DEGs lists. This seems to confirm the finding in an independent study using TCGA RNA-Seq data, where perilipin-1 (PLIN1) mRNA expression is found to be significantly downregulated in human breast cancers [28]. LEP, another downregulated genes among both DEGs lists, is an important regulator of adipose tissue mass. Leptin, the protein product the LEP gene, binds to leptin receptor to activate downstream pathways to inhibit feeding and promote energy expenditure. The disruption on (or resistance to) the action of leptin is a hallmark of obesity, which in turn is a strong risk factor for several diseases including diabetes, cardiovascular disease, and certain types of cancers [29]. Recently, two independent studies reported that LEP was among the most down-regulated genes in breast cancers of Lebanese [30] and Saudi Arabian cohorts [31].

We also performed KEGG human pathway enrichment analysis on the set of genes (probes) from the differential expression analysis. Additional files 6 and 7 list all the KEGG pathways examined and their statistical significance, for the Tumor-vs-Normal 415-probe gene signature and the Tumor-vs-Adjacent 164-probe gene signature respectively. Commonly enriched KEGG human pathways include PPAR signaling pathway, Adipocytokine signaling pathway, AMPK signaling pathway, ECM-receptor interaction, Tyrosine metabolism, Drug metabolism - cytochrome P450, Malaria, Fatty acid biosynthesis, and Histidine metabolism. It is interesting to note that the roles of PPAR signalling in cancer has been well documented in the literature [32, 33], and recently there is evidence to suggest that PPAR signaling pathway may be an important predictor of breast cancer response to neoadjuvant chemotherapy [34], and the activation of PPAR beta can inhibit human breast cancer cell line tumorigenicity. Similarly the AMPK signaling pathway has also been implicated in cancers [35–37], and there has been significant research interest to target AMPK for cancer prevention and treatment [38].

### Gene signatures and connectivity mapping

From the Tumor-vs-Normal differential gene expression analysis, 415 gene probes were selected as both statistically and biological significant. While theoretically it was possible to include all these 415 genes into a single gene signature to perform connectivity mapping, a gene signature of this length would return a very long list of candidate drugs all connected to the gene signature someway or another. While the connections to these drugs would be real reflection of some aspects of the biology contained in the gene signature, the danger is that with a large number of drugs returned, the key biological message could be well buried into much fine details and thus dilute the prominence of the key biological processes. On a technical side, a gene signature with 415 genes is too long to be handled efficiently by the QUADrATiC system because of the computational demands. To achieve a feasible connectivity mapping analysis and also to increase the robustness of the results obtained, we adopted a different strategy tackling this problem. The idea is that our confidence in the connectivity mapping results is increased when non-overlapping gene signatures of the same biological states can return significant overlaps among the candidate drugs. This is possible, because these non-overlapping gene signatures capture different aspects of the same biological states. In our analysis we divided the 415 genes into 5 non-joint sets of genes, 83 genes per set, as determined by the following process. First these 415 gene were ordered by combined ranking based on their *p*-values and fold changes. Then the genes at positions 1,6,11,16, ⋯,411 form the first set; similarly the genes at positions 2,7,12,17, ⋯, and 412 form the second set; and so on and so that the last set of genes include those at position 5,10,15,20, ⋯, and 415. In this way, we constructed 5 separate gene signatures for the Tumor vs Normal comparison, and each consisting of a set of equal distanced genes on the ordered list of 415 significant genes. The distance between two consecutive genes is simply the number of distinct gene signatures to be constructed, which in the case of Tumor vs Normal is 5. In general, gene signature i consists of genes at the positions i, i+k, i+2k, i+3k, ⋯ i+(n-1)k, where *k* is the number of distinctive gene signatures to compile, and n is the number of genes to be included in each gene signature. For the Tumor vs Normal analysis, *k*=5, *n*=83. The full list of these 415 genes can be found in Additional file 4; and in Additional file 8 the 5 separate lists of 83 genes are included, with each list consisting of genes equally distanced in their ranks. We then used each gene list as a signature to query the core drug reference database, and returned FDA drugs that were significantly connected to the signature. If a drug turned out to be significantly connected to all (or most) of those separate breast cancer gene signatures, we would have much increased confidence in this drug. We observed that non-overlapping gene signatures returned overlapping drugs, which were then further examined on their directions of association with breast cancer risk (increase or reduce), and their overall connection scores.

Connectivity mapping using these five gene signatures resulted in five separate lists of drugs with their connection scores and *p*-values obtained. These five lists of drugs were combined and only the drugs that were significant for at least 3 out the 5 signatures were selected for further analysis. Furthermore, the connection scores for any selected drugs must have the same sign across all 5 gene signatures. This ensured that the selected drugs all have consistent directions of actions. Table 2 includes the drugs with significant connections in all these five input gene signatures. Additional file 9 provides a longer list of top drugs, including significant drugs in at least three out of five input gene signatures. Drugs which appeared significant multiple times from different gene signatures were considered to be very strong candidates representing strong association with the disease state. Z-scores indicate the direction of effects that the drug could exert on the gene signature (hence the breast cancer disease state). A positive z-score indicates the increased risk of the drug on developing breast cancer whereas a negative z-score indicates the treatment path. We were looking for drugs that may alter the risk of breast cancer development, in this instance we found that a few top drugs with negative z-scores are known to be used for treating cancers. In particular, among the 26 compounds listed in Table 2 with negative z-score, 14 of them are known anti-cancer drugs. These are: cytarabine (mean z score = -7.09), gemcitabine (-6.55), methotrexate (-6.81), topotecan (-5.85), etoposide (-5.99), doxorubicin (-4.76), amethopterin (-6.24), S1025 (-5.97), teniposide (-5.01), 2-chloro-2’-deoxyadenosine (-4.43), azacitidine (-5.16), aminolevulinic acid (-4.98), chlorambucil (-4.46), and S1222 (-3.82). This increases the confidence on the results obtained and moreover confirms the study has been in the right direction. In the other direction of action, 7 out of 33 compounds listed in Table 2 have positive z-scores, and therefore, they are candidate drugs predicted to increase breast cancer risk. These 7 drugs are: sulfafurazole (mean z score = 6.26), dihomo-gamma-linolenic acid (6.03), minoxidil (5.75), cefotiam hydrochloride (5.33), sulfacetamide (5.11), 9-cis retinoic acid (5.11), and doxylamine succinate (4.59). The number in the parenthesis following the drug name is the mean connectivity z score as obtained from the QUADrATiC connectivity mapping analysis. We searched these 7 drugs against the list of Known and Probable Human Carcinogens [39] developed by the International Agency for Research on Cancer (IARC) and the US National Toxicology Program (NTP), but they were not found among the carcinogens list. Their absence from the list of known carcinogens however does not mean that our predictions are wrong. It may simply reflect the fact that these drugs are approved medications still in use and their potential carcinogenesis property (as suggested by our study) is not known yet. Further discussions on a few of these drugs are provided in the Discussion section to suggest possible mechanistic explanations why they could increase breast cancer risk.

**Table 2:** Combined results of the significant drugs returned from sscMap using the 5 Tumor-vs-Normal gene signatures as queries

Compound	Replicates	Mean z	*p*_1_	*z*_1_	*p*_2_	*z*_2_	*p*_3_	*z*_3_	*p*_4_	*z*_4_	*p*_5_	*z*_5_
budesonide	85	-7.78	2.00E-09	-6	1.20E-13	-7.41	1.70E-20	-9.28	9.60E-12	-6.81	6.00E-21	-9.39
menadione	364	-7.26	4.50E-12	-6.92	8.20E-18	-8.6	2.60E-10	-6.32	1.20E-12	-7.1	1.90E-13	-7.35
cytarabine	48	-7.09	8.70E-16	-8.04	2.00E-20	-9.26	5.90E-11	-6.55	3.30E-06	-4.65	3.80E-12	-6.95
methotrexate	10	-6.81	4.20E-11	-6.6	1.30E-18	-8.8	2.40E-09	-5.97	2.80E-09	-5.94	1.80E-11	-6.72
gemcitabine hydrochloride	107	-6.55	7.30E-12	-6.85	8.10E-20	-9.11	1.30E-10	-6.43	3.60E-06	-4.63	1.00E-08	-5.72
milnacipran	37	-6.39	1.40E-07	-5.26	5.10E-13	-7.22	7.90E-15	-7.77	2.20E-05	-4.24	1.00E-13	-7.44
sulfafurazole	34	6.26	2.60E-10	6.32	1.20E-08	5.7	6.50E-11	6.53	8.10E-05	3.94	1.60E-18	8.78
amethopterin	36	-6.24	2.20E-07	-5.19	7.10E-19	-8.87	8.00E-12	-6.84	2.20E-04	-3.69	4.30E-11	-6.59
dihomo-gamma-linolenic acid	52	6.03	3.50E-10	6.28	1.10E-06	4.88	8.80E-10	6.13	1.60E-05	4.32	1.10E-17	8.57
etoposide	35	-5.99	6.20E-08	-5.41	1.60E-20	-9.28	5.30E-07	-5.02	8.00E-07	-4.93	1.10E-07	-5.31
s1025	65	-5.97	5.80E-07	-5	1.60E-11	-6.74	1.70E-05	-4.3	3.00E-08	-5.54	1.50E-16	-8.25
auranofin	3	-5.92	2.90E-09	-5.94	2.00E-11	-6.7	1.20E-06	-4.85	1.10E-09	-6.1	1.70E-09	-6.02
topotecan hcl	23	-5.85	1.20E-09	-6.08	8.40E-11	-6.49	1.40E-06	-4.82	6.80E-07	-4.97	6.20E-12	-6.87
minoxidil	88	5.75	1.90E-09	6.01	2.40E-04	3.67	1.10E-11	6.79	1.20E-05	4.38	2.50E-15	7.92
dlotrimazole	47	-5.6	5.50E-09	-5.83	5.20E-11	-6.57	8.80E-07	-4.92	2.60E-06	-4.7	2.10E-09	-5.99
metaraminol bitartrate	10	-5.53	2.50E-09	-5.96	6.30E-06	-4.52	8.10E-16	-8.05	2.60E-05	-4.2	9.60E-07	-4.9
cefotiam hydrochloride	33	5.33	3.00E-10	6.3	7.30E-08	5.38	1.10E-06	4.88	1.90E-04	3.74	2.20E-10	6.34
azacitidine	12	-5.16	5.00E-05	-4.05	6.80E-11	-6.52	2.90E-07	-5.13	2.30E-07	-5.18	8.70E-07	-4.92
sulfacetamide	90	5.11	3.70E-06	4.63	3.50E-08	5.52	1.90E-07	5.21	2.10E-04	3.71	8.10E-11	6.5
9-cis retinoic acid	22	5.11	9.80E-07	4.9	7.80E-09	5.77	6.80E-08	5.4	1.60E-04	3.77	1.00E-08	5.73
teniposide	347	-5.01	8.30E-06	-4.46	2.50E-15	-7.91	1.60E-04	-3.77	4.70E-06	-4.58	1.30E-05	-4.36
aminolevulinic acid	44	-4.98	5.40E-05	-4.04	2.60E-10	-6.32	5.00E-05	-4.05	7.30E-04	-3.38	1.10E-12	-7.12
fluvastatin	107	-4.93	1.30E-04	-3.82	1.20E-10	-6.44	1.40E-06	-4.82	1.10E-05	-4.4	2.70E-07	-5.14
doxorubicin	159	-4.76	7.10E-08	-5.39	2.50E-09	-5.96	4.80E-04	-3.49	7.70E-05	-3.95	5.90E-07	-4.99
mometasone furoate	29	-4.74	1.40E-05	-4.35	3.00E-07	-5.12	4.80E-05	-4.06	1.90E-05	-4.27	4.00E-09	-5.88
desipramine hydrochloride	57	-4.61	1.60E-05	-4.32	3.00E-05	-4.17	8.30E-06	-4.46	9.30E-06	-4.43	1.60E-08	-5.65
doxylamine succinate	57	4.59	9.10E-07	4.91	1.30E-04	3.83	1.50E-05	4.33	2.30E-05	4.24	1.50E-08	5.66
sertraline hydrochloride	46	-4.55	9.60E-05	-3.9	2.00E-05	-4.27	1.70E-07	-5.23	2.20E-04	-3.69	1.60E-08	-5.65
diloxanide furoate	58	-4.52	4.80E-07	-5.03	3.50E-05	-4.14	9.80E-07	-4.9	3.80E-05	-4.12	1.00E-05	-4.41
chlorambucil	166	-4.46	8.60E-05	-3.93	4.50E-09	-5.87	1.80E-06	-4.77	1.50E-05	-4.33	7.10E-04	-3.39
2-chloro-2’-deoxyadenosine	49	-4.43	1.50E-05	-4.32	7.70E-08	-5.37	9.40E-06	-4.43	5.60E-04	-3.45	4.60E-06	-4.58
bacitracin	11	-4.11	8.10E-05	-3.94	9.30E-08	-5.34	4.00E-04	-3.54	1.30E-04	-3.82	8.70E-05	-3.92
s1222	66	-3.82	3.80E-04	-3.55	2.20E-06	-4.73	2.80E-04	-3.63	4.60E-04	-3.51	2.40E-04	-3.68

This table lists only those drugs that are significant for all these 5 signatures

From the Tumor-vs-Adjacent differential gene expression analysis, 164 gene probes were selected as both statistically and biologically significant. Following a similar procedure as described above, we divided these 164 significant genes into 4 distinctive gene signatures, with the parameters *k*=4 and *n*=41. The full list of these 164 significant genes and their split into 4 non-joint gene signatures are provided in Additional file 5 and Additional file 10, respectively. These gene signatures were then used as input to the connectivity mapping process separately and the results were combined to obtain the final list of drugs. Additional file 11 provides a list of the top drugs from this batch of connectivity mapping analysis, which includes significant drugs in at least three out of four input gene signatures.

Comparing the significant drugs obtained using the Tumor-vs-Normal gene signatures and those using Tumor-vs-Adjacent gene signatures, again there is a big overlap between the two sets of significant drugs, 146 drugs for Tumor-vs-Normal, and 39 drugs for Tumor-vs-Adjacent, which are listed in Additional files 9 and 11 respectively. In particular, 35/39=90*%* of drugs returned using the Tumor-vs-Adjacent gene signatures are included in the results obtained using the Tumor-vs-Normal gene signatures. This probably reflects the fact that there is a big overlap of genes between the Tumor-vs-Normal 415-probe and Tumor-vs-Adjacent 164-probe gene signatures, as described in previous sections.

From the Adjacent-vs-Normal differential expression analysis, only 4 genes qualified through the filtering criteria and were selected as both statistically and biological significant. This result suggests that the difference between the two groups are not significant enough and the two states could be considered as one. No further analysis was performed based on this result.

### Comparison to standard CMap02

**Fig. 6:**
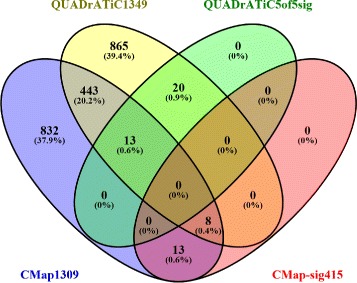
Comparison of the numbers of compounds in the CMap and QUADrATiC systems, and the numbers of significant drugs returned using the 5 disjoint signatures with QUADrATiC and that using a single 415-gene signature with CMap. Blue ellipse: The set of small molecule compounds that were used to generate the CMap reference gene expression profiles. Red ellipse: The set of CMap compounds that are significantly connected to the single 415-gene signature as obtained from CMap; Yellow ellipse: The set of FDA approved drugs that are included in the reference gene expression profiles of QUADrATiC; Green ellipse: The top ranked FDA approved drugs that are significantly connected to all 5 sub-signatures used to query QUADrATiC. This is a set of drugs listed in Table 2

The standard CMap approach does not deal with how a query gene signature is created, but simply accepts a list of selected gene probes (with their up or down regulation status) as the input, however the probes were selected. For comparison, we also carried out an analysis using the standard CMap approach, ie, Querying the CMap02 [40] with the 415 gene probes as a single input signature. The results are present in Table 3. Figure 6 provides a Venn diagram comparing the sets of compounds in the CMap and QUADrATiC systems, and also the sets of significant drugs returned using the 5 disjoint 83-gene signatures with QUADrATiC and that using a single 415-gene signature with CMap. As can be ascertained from this figure, between the CMap collection of reference profiles (for 1309 small molecule compounds) and the QUADrATiC collection of reference profiles (for 1349 FDA approved drugs), there are 464 common compounds. Out of these 464 common drugs, the standard CMap approach returned 8 significant drugs. These are: phenoxybenzamine (CMap mean score = -0.816), guanabenz (0.63), podophyllotoxin (0.741), tranexamic-acid (0.616), levonorgestrel (-0.638), nadolol (0.715), chlorpromazine (-0.494), and medrysone(-0.658). The number in the parenthesis following the compound name is the mean connectivity score as obtained from the CMap02 web server. Note that the mean scores from CMap02 are not to be compared with the connectivity z scores from QUADrATiC; the signs of both types of scores nevertheless are comparable. For those 4 compounds with negative CMap mean scores, phenoxybenzamine, levonorgestrel, chlorpromazine, and medrysone, there is currently no literature evidence to suggest they have anti-cancer properties.

**Table 3:** The results of the significant drugs returned from querying the original CMap02 (https://portals. broadinstitute.org/cmap/) using the Tumor-vs-Normal 415-gene signature as input

Rank	Compound name	Mean	n	Enrichment	p	p*N (=929)	FDR
1	adiphenine	0.813	5	0.958	0	0	0
2	resveratrol	-0.752	9	-0.857	0	0	0
3	genistein	0.331	17	0.57	0	0	0
4	trichostatin A	-0.388	182	-0.264	0	0	0
5	aciclovir	0.65	6	0.853	0.00002	0.019	0.004
6	0175029-0000	-0.67	6	-0.819	0.0001	0.093	0.015
7	etiocholanolone	0.598	6	0.794	0.00018	0.167	0.024
8	guanabenz	0.63	5	0.841	0.00024	0.223	0.028
9	phenoxybenzamine	-0.816	4	-0.892	0.00026	0.242	0.027
10	nadolol	0.715	4	0.881	0.00028	0.260	0.026
11	podophyllotoxin	0.741	4	0.881	0.00028	0.260	0.024
12	pHA-00745360	0.504	8	0.682	0.00038	0.353	0.029
13	felbinac	0.711	4	0.869	0.0004	0.372	0.029
14	meticrane	-0.663	5	-0.822	0.00042	0.390	0.028
15	levonorgestrel	-0.638	6	-0.746	0.00052	0.483	0.032
16	prestwick-1103	0.691	4	0.857	0.00056	0.520	0.033
17	8-azaguanine	-0.765	4	-0.865	0.00062	0.576	0.034
18	dL-thiorphan	-0.821	2	-0.983	0.00068	0.632	0.035
19	tranexamic acid	0.616	5	0.802	0.0007	0.650	0.034
20	medrysone	-0.658	6	-0.726	0.00085	0.790	0.039
21	chlorpromazine	-0.494	19	-0.429	0.001	0.929	0.044

Out of the same set of 464 common drugs, the new approach developed here returned 13 significant drugs. These are: minoxidil (mean z = 5.75), bacitracin (-4.11), methotrexate (-6.81), fluvastatin (-4.93), azacitidine (-5.16), chlorambucil (-4.46), doxorubicin (-4.76), etoposide (-5.99), sulfafurazole (6.26), clotrimazole (-5.6), sulfacetamide (5.11), budesonide (-7.78), and menadione (-7.26). There are no overlap between these 13 drugs and those 8 drugs from CMap02 above, but the new approach picked up some drugs already known to be anti-cancer drugs (methotrexate, azacitidine, chlorambucil, doxorubicin, and etoposide, which are briefly discussed in the “Discussion” section), and importantly their connection scores were all negative. This demonstrates that the new approach picked up compounds that are confirmed relevant to the current study. On the other hand, the standard CMap approach picked a few drugs that are known to have anti-cancer effects or have been investigated for such properties, eg, resveratrol (-0.752) and trichostatin A (-0.388). But these drugs are not represented in the QUADrATiC because they are not already FDA approved. This indicates that both approaches can be used complementarily to each other. For the purpose of screening medications (which have to be approved drugs), the approach developed here is more appropriate.

### Comparison to random division of sub-signatures

**Fig. 7:**
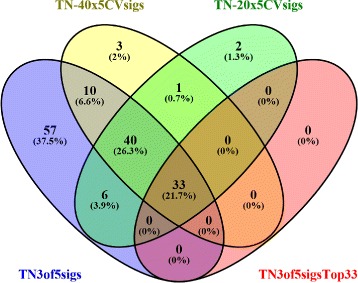
Comparison of significant drugs obtained from the equal-distance division sub-signatures versus those from systematic 5-fold random division sub-gene signatures. Blue ellipse: The set of drugs that are significantly connected to 3 out of 5 of equal-distance division sub-signatures in the connectivity mapping analysis. Red ellipse: The top 33 drugs from the equal-distance division method of sub-signatures. This is a set of drugs reported in Table 2; Green ellipse: Result obtained from 20 repeats of the random division of sub-signatures. Enclosed in this ellipse are drugs that are significant in 19 out of the 20 runs (95%); Yellow ellipse: Result obtained from another 40 repeats of the random division of sub-signatures. Enclosed in this ellipse are drugs that are significant in 38 out of the 40 runs (95%)

With regard to the creation of non-overlapping gene signatures from the combined list of ranked DEGs, here we adopted the method of an equal-distanced partition of these ranked candidate genes. There could be other ways of partitioning the candidate DEGs, for example, by random division of the DEGs into *k* sub-signatures. Of course, the equal-distance partition method is only a special realization of the more general random partition method. In principal, when the random division method is repeated many times, the overall results would be more sytematic and less dependent on any one specific division of the DEGs. However, for each random division of the DEGs, the connectivity mapping analyses still need to be conducted using our QUADrATiC system. The amount of manual input involved and the computational loads in each run prohibited us from adopting this fully systematic approach. Nevertheless, we did perform a small number of runs of the random partition of DEGs followed by connectivity mapping. With the same procedure as for the equal-distance partitioned sub-signatures, each run with a random division of sub-signatures provided list of drugs that are significantly connected to at least 3 out 5 sub-signatures. The drugs are then ranked according to their percentage of times they are significant in those individual runs. The top drugs returned this way had a significant overlap with the ones from the equal-distance division method. As can be seen from Fig. 7, the equal distance division method returned 146 significant drugs (represented by the blue ellipse labeled as TN3of5sigs in the figure); while random division of sub-signatures method with 20 repeats (TN-20x5CVsigs) returned 82; and with 40 repeats (TN-40x5CVsigs) the number of significant drugs returned is 87. First of all, the overlap between the two series of random division runs is 74/82=90*%* of the TN-20x5CVsigs results or 74/87=85*%* of TN-40x5CVsigs results, suggesting that these results are quite stable although they were different realizations of random division runs. Secondly, except for a small number of drugs (3 in the case of TN-20x5CVsigs, or 4 in the case of TN-40x5CVsigs), almost all those 82 (or 87) significant drugs returned from the random-division method TN-20x5CVsigs (TN-40x5CVsigs) were part of the 146 significant drugs returned by the equal-distance division method TN3of5sigs. This suggests that the equal-distance partition method gives results that are highly consistent with that from the more systematic but more expensive random division methods. So on balance, the equal-distance division methods seems to provide a feasible and reliable solution. Furthermore, those top ranked drugs from the equal distance-division method (TN5of5sigs, 33 drugs) are all identified by both series of the TN-20x5CVsigs and TN-40x5CVsigs runs with no exception, therefore corroborating their high ranks among the 146 drugs from TN3of5sigs. Taken together, the various novel procedures developed in this study greatly enhanced our confidence in the final significant drugs obtained.

## Discussion

Previously in our research effort on gene expression connectivity mapping and its application, we developed several techniques to enhance the robustness of the results of the drugs returned, for example, the gene signature perturbation approach developed in [41] and the gene signature progression approach developed in [42]. As compared to the gene signature perturbation approach, the procedure implemented in this work represents very different strategy to increase the robustness of the results. The input gene signatures in the perturbation approach were mostly similar; in fact, any two input gene signatures in that approach only differed by one single gene probe, hence the term “perturbation” (only minor/small change; and keeping the overall original shape). The approach in this paper, however, is dramatically different. There is no single gene overlaps between any input gene signatures used in the current approach. Yet they can still return a significant number of common drugs. This is possible because of the underlying biology, and these input gene signatures simply reflect different aspects of the same biological state or process.

The top significant drugs listed in Table 2 includes 26 with negative connectivity z scores, suggesting they have potential to suppress the breast cancer disease state. Reassuringly, 14 out of these 26 are already known to be anti-cancer drugs, providing strong evidence to support the validity of the findings here. It is interesting to note that 5 of these 14 anti-cancer drugs: methotrexate, azacitidine, chlorambucil, doxorubicin, and etoposide are actually present in the CMap02 collection of reference profiles, but they were not picked up by the standard CMap approach as significant drugs. This shows the unique value provided by the new approach developed in this paper. Here we briefly discuss a few of these significant drugs returned.

Methotrexate is one of the most widely studied therapeutics agents, an antineoplastic antimetabolite with immunosuppressant properties. It is effective to treat autoimmune diseases such as rheumatoid arthritis and many types of cancers [43]. Methotrexate is known to interferes with folate metabolism, mainly through inhibiting folic acid reductase, leading to inhibition of DNA synthesis and cellular replication, to exert its anti-tumor activity [44]. There is also evidence to suggest that methotrexate may additionally exert its anti-cancer activities through other molecular targets, such as the inhibition of histone deacetylase (HDAC) [45]. Methotrexate is widely used in chemotherapy, either alone or in combination with other agents for the treatment of a number of cancers including breast cancer, lung cancer and leukemia.

Azacitidine is a chemical analog of cytidine, a pyrimidine nucleoside in DNA and RNA. This drug is approved in the USA for the treatment of all subtypes of myelodysplastic syndrome (MDS) [46] and is approved in many other countries (eg the European Union and Australia) for AML (Acute Myeloid Leukaemia) patients not eligible for a stem cell transplant [47]. Azacitidine inhibits DNA methyltransferase, causing DNA hypomethylation, which in turn may restore normal function of aberrantly silenced tumor suppressor genes, underlying azacitidine’s antileukemic activity [48]. Azacitidine may also exert its antileukemic effects by causing direct cytotoxicity on abnormal hematopoietic cells in the bone marrow, through its incorporation into cellular nucleic acid [49], leading to inhibition of protein synthesis, DNA damage, and cell death.

Among the list of 33 compounds in Table 2, the 7 compounds with positive connectivity z-scores are: sulfafurazole, cefotiam hydrochloride, sulfacetamide, 9-cis retinoic acid, minoxidil, doxylamine succinate, and dihomo-gamma-linolenic acid. Of these 7 compounds, sulfafurazole, cefotiam hydrochloride, and sulfacetamide are anti-bacterial agents that are used to treat various bacterial infections. Currently there is no reported studies investigating their carcinogenic effect. 9-cis retinoic acid (also known as alitretinoin) is an active metabolite of vitamin A. It is approved by FDA for topical treatment of cutaneous lesions in patients with AIDS-related Kaposi’s sarcoma. This compound binds to and activates intracellular retinoid receptors, which then act as transcription factors, to control the process of cellular differentiation and proliferation in both normal and neoplastic cells [50]. In recent year, 9-cis retinoic acid and its isomer all-trans retinoic acid have been investigated mainly as therapeutic agents for different types of cancers, including human breast cancer [51–53]. Further research is needed to gain a better understanding why this compound consistently attained positive connectivity scores with the breast cancer signatures in the current study.

Minoxidil is a compound used in regrowing gradually thinning or loss hair, especially a hair growth product after chemotherapy. The relationship between the use of minoxidil and the risk of developing breast cancer has already been an active topic of discussion. Minoxidil is a potassium channel opener or activator [54], where potassium channels are known to play a key role in breast cancer proliferation [55]. In particular there were in vitro evidence to show that minoxidil as a potassium channel opener stimulated growth of MCF-7 human breast cancer cells [56] as well as PC3 human prostate cancer cells [57]. Taken together, these might point to a possible mechanistic explanation why minoxidil could be an enhancing factor for breast cancer development as indicated by the connectivity mapping results in the current study.

Doxylamine succinate is a first-generation antihistamine used to relieve symptoms of allergy, hay fever, and the common cold. Notably doxylamine in combination with vitamin B6 (pyridoxine) is prescribed to prevent morning sickness in pregnant women. In recent years, there has been some discussion on whether doxylamine-pyridoxine should continue to be used for nausea and vomiting of pregnancy because some conflicting evidence links doxylamine-pyridoxine use to pyloric stenosis and childhood malignancies [58].

Dihomo-gamma-linolenic acid (DGLA) is an uncommon fatty acid used for nutritional supplementation and for treating dietary shortage or imbalance. DGLA has been shown to reduce the production/activity of tumor necrosis factor alpha (TNF *α*) [59], while TNF *α* is implicated in both apoptosis and cell proliferation, thus having a paradoxical role in anti-cancer activity and tumor promotion [60]. DGLA is made in the body by the elongation of gamma-linolenic acid (GLA). Evening primrose oil (EPO) contains high amounts of GLA, which has traditionally been used for a range of ailments, commonly premenstrual and menopausal symptoms in women, particularly breast pain, and some skin disorders such as eczema despite the lack of evidence for its effectiveness in such disorders [61, 62].

The few compounds discussed here are used for various purposes, though they are prone to uses by women at different stages of their lives, which makes it particularly relevant to investigate the potential impact of these drugs in the development of breast cancer.

## Conclusions

In this work, we developed an integrated meta-analysis approach to screening medications for their potentials to alter disease risks through connectivity mapping, using breast cancer as a case study. This approach involved unified processing and normalization of raw gene expression data, systematic removal of batch effects, and multiple runs of balanced sampling for differential expression analysis, which provided high quality inputs to subsequent connectivity mapping analysis. There, our novel idea was that non-overlapping gene signatures returning overlapping significant drugs was a confidence booster of the connectivity mapping results and also a confirmation of the quality and relevance of the input gene signatures. This was underpinned by the fact that those non-joint gene signatures actually represented different aspects of the same biological states, and hence enabled them to retrieve from connectivity mapping separate lists of candidate drugs with significant overlaps. Consequently, we can have high confidence in the top drugs’ predicted effects on breast cancers. Of particular note, among the top 26 compounds identified as inversely connected to breast cancer, 14 of them are known anti-cancer drugs. A few candidate drugs with potential to enhance breast cancer or increase the risk of the disease were also identified; further investigation on a large population is required to firmly establish their effects on breast cancer risks. In conclusion, this work presents novel ideas for the creation of gene signatures and for connectivity mapping analysis, and provides a paradigm for identifying medications with potential to alter cancer risks through gene expression connectivity mapping.

## Additional files

Additional file 1 Breast cancer datasets. Summary Tables of GEO datasets retrived and selected for inclusion into the current study. (XLS 2931 kb)

Additional file 2 Power and sample size. A description of the methods used for the calculation of power and sample size for gene differential expression analysis. (DOCX 13 kb)

Additional file 3 Power and sample size table. Results of power and sample size calculations as obtained using the procedure described in Additional file 2. (XLSX 14 kb)

Additional file 4 Tumor vs Normal differentially expressed genes. The full list of 415 significant genes selected from Tumor-vs-Normal differential expression analysis. (TAB 36 kb)

Additional file 5 Tumor vs Adjacent differentially expressed genes. The full list of 164 significant genes selected from Tumor-vs-Adjacent differential expression analysis. (TAB 14 kb)

Additional file 6 Tumor vs Normal 415-gene signature KEGG pathways. Full results of the KEGG human pathway enrichment analysis on the set of 415 significant genes selected from Tumor-vs-Normal differential expression analysis. (XLSX 121 kb)

Additional file 7 Tumor vs Adjacent 164-gene signature KEGG pathways. Full results of the KEGG human pathway enrichment analysis on the set of 164 significant genes selected from Tumor-vs-Adjacent differential expression analysis. (XLSX 112 kb)

Additional file 8 Tumor vs Normal 5 sub-gene signatures. The 5 non-joint 83-gene signatures are included, each consisting of genes equally distanced in the rankings of the 415 significant genes from Tumor vs Normal differential expression analysis. (XLSX 40 kb)

Additional file 9 Significant drugs for Tumor vs Normal 5 gene signatures. The list of candidate drugs from connectivity mapping analysis using the 5 Tumor-vs-Normal gene signatures. List drugs are significant in at least three out of five input gene signatures. (XLSX 108 kb)

Additional file 10 Tumor vs Adjacent 4 gene signatures. The 4 non-joint 41-gene signatures are included, each consisting of genes equally distanced in the rankings of the 164 significant genes from Tumor vs Adjacent differential expression analysis. (XLSX 21 kb)

Additional file 11 Significant drugs for Tumor vs Adjacent 4 gene signatures. The list of candidate drugs from connectivity mapping analysis using the 4 Tumor-vs-Adjacent gene signatures. List drugs are significant in at least three out of four input gene signatures. (XLSX 19 kb)

## Abbreviations

ALL: Acute lymphoblastic leukemia
AML: Acute myeloid leukemia
CMap: Connectivity map
CMap02: Connectivity map build 02
DEG: Differentially expressed gene
DGE: Differential gene expression
eFDR: empirical false discovery rate
FDA: Food and drug administration
FDR: False discovery rate
GEO: Gene expression omnibus
HDAC: Histone deacetylase
LINCS: Library of network-based cellular signatures
MAS5: Microarray suite 5.0
MDS: myelodysplastic syndrome MIAME: Minimum information about microarray experiment
PCA: Principal component analysis
