# Supplementary material for: An integrated meta-analysis approach to identifying medications with potential to alter breast cancer risk through connectivity mapping
Source: BMC Bioinformatics. 2017 Dec 21;18:581. doi: 10.1186/s12859-017-1989-x (PMC5740937; doi:10.1186/s12859-017-1989-x)
Supplement: Supplementary file 2 — Power and sample size. A description of the methods used for the calculation of power and sample size for gene differential expression analysis. (DOCX 13 kb) [file 12859_2017_1989_MOESM2_ESM.docx]

Supplementary File 02:

**Power and sample size calculations for differential gene expression analysis:**

For power and sample size calculations, we first estimated the expression variance of all the genes using the data matrix. With the log2Mas5 gene expression measure, the median standard deviation of control samples is 0.87, while the breast cancer group is 0.85. We used 0.87 as a representative standard deviation in our subsequent calculations, in the knowledge that the power will be the median power. We performed a series of power calculations: at the sample size n=100 per group, two-sample Z test gives almost full power (power approaching 1) to detect a differential gene expression of delta=2, at the significance level of alpha = 4.5e-5. This significance level is the actual threshold p-value we used in the differential expression analysis, which is 1/22277 = 4.5e-5. On the other hand, in our actual differential expression analysis, we used two-sample Wilcoxon test. The power calculation method for Wilcoxon test is not yet well developed or readily available as for Z test. So we used the powers from Z test as estimates. The non-parametric Wilcoxon test is generally more robust than its parametric counterpart Z or T tests, because the Wilcoxon test does not require the assumption of normal distribution of expression values. The cost of this increased robustness of Wilcoxon test is that it is not as powerful as T or Z test when the normal distribution is true. But since we are working with sample sizes that give almost full power for Z test, the expected slight reduction in actual power by using the Wilcoxon test is not a matter of concern.

Supplementary file 03 provides a table of sample sizes and corresponding power. As can been seen from this table, if we assume that 95% of gene are non-differentially expression (the pi0=0.95), the overall false discovery rate achievable 0.0009, much lower than the commonly used tolerable FDR of 0.05.

With the achievable power and FDR as described above, our study here provides us with the ultimate statistical confidence in the candidate genes identified for subsequent gene signature constructions.
